# VGluT1 Deficiency Impairs Visual Attention and Reduces the Dynamic Range of Short-Term Plasticity at Corticothalamic Synapses

**DOI:** 10.1093/cercor/bhz204

**Published:** 2019-11-11

**Authors:** Sarah H Lindström, Sofie C Sundberg, Max Larsson, Fredrik K Andersson, Jonas Broman, Björn Granseth

**Affiliations:** Department of Clinical and Experimental Medicine, Division of Neurobiology, Linköping University, Linköping, 58185, Sweden

**Keywords:** facilitation, feedback, lateral geniculate nucleus, modulatory synapse, patch-clamp electrophysiology

## Abstract

The most common excitatory neurotransmitter in the central nervous system, glutamate, is loaded into synaptic vesicles by vesicular glutamate transporters (VGluTs). The primary isoforms, VGluT1 and 2, are expressed in complementary patterns throughout the brain and correlate with short-term synaptic plasticity. VGluT1 deficiency is observed in certain neurological disorders, and hemizygous (VGluT1^+/−^) mice display increased anxiety and depression, altered sensorimotor gating, and impairments in learning and memory. The synaptic mechanisms underlying these behavioral deficits are unknown. Here, we show that VGluT1^+/−^ mice had decreased visual processing speeds during a sustained visual-spatial attention task. Furthermore, in vitro recordings of corticothalamic (CT) synapses revealed dramatic reductions in short-term facilitation, increased initial release probability, and earlier synaptic depression in VGluT1^+/−^ mice. Our electron microscopy results show that VGluT1 concentration is reduced at CT synapses of hemizygous mice, but other features (such as vesicle number and active zone size) are unchanged. We conclude that VGluT1-haploinsuficiency decreases the dynamic range of gain modulation provided by CT feedback to the thalamus, and this deficiency contributes to the observed attentional processing deficit. We further hypothesize that VGluT1 concentration regulates release probability by applying a “brake” to an unidentified presynaptic protein that typically acts as a positive regulator of release.

## Introduction

Glutamate is the most common excitatory neurotransmitter in the central nervous system. This amino acid is loaded into small synaptic vesicles by vesicular glutamate transporters (VGluTs). Three VGluT isoforms (VGluT1–3) are expressed in the brain (Bellocchio et al. 2000; Fremeau et al. 2001, 2002; Gras et al. 2002). VGluT1 and 2 are expressed in a largely complementary pattern at glutamatergic synapses (Fremeau et al. 2004; Wojcik et al. 2004), while VGluT3 (and sometimes 2) can be found at synapses dominated by other neurotransmitters (Gras et al. 2002; Descarries et al. 2008; Marshak 2016; Kljakic et al. 2017). VGluT2 is most typically expressed by synapses from the thalamus, brainstem, or spinal cord. These synapses display high probability of transmitter release (P_R_) and short-term depression. VGluT1 is usually expressed at synapses from neocortex, cerebellum, or hippocampus. These synapses are characterized by low P_R_ and short-term facilitation (Fremeau et al. 2001; Granseth and Lindström 2003; Sherman and Guillery 2011; Balaram et al. 2013).

Altered VGluT1 expression levels have been detected in cortical and hippocampal samples from schizophrenic, Alzheimer’s, and Parkinson’s disease patients (Eastwood and Harrison 2005; Kashani et al. 2007, 2008). Knockout mice with homozygous suppression of VGluT1 expression (VGluT1^−/−^) develop a progressive phenotype of blindness, uncoordinated movement, and failure to thrive starting at 2–3 weeks of age (Fremeau et al. 2004; Wojcik et al. 2004). These severe manifestations are absent from hemizygous (VGluT1^+/−^) mice (Wojcik et al. 2004; Balschun et al. 2010; Granseth et al. 2015), in which VGluT1 expression is only reduced to about 60% of normal (Tordera et al. 2007). However, a number of milder behavioral impairments have been identified. VGluT1^+/−^ mice show signs of increased anxiety- and depressive-like behavior (Tordera et al. 2007) and altered sensorimotor gating (Fremeau et al. 2004; Inta et al. 2012). Deficits in learning and memory are more complex: Visual and spatial learning and memory are normal, but impairments have been observed in working memory, object (and social) recognition, and reversal learning (Tordera et al. 2007; Balschun et al. 2010; Inta et al. 2012; Granseth et al. 2015). The neuronal mechanisms that mediate the behavioral deficits observed in VGluT1^+/−^ mice are unknown.

Experiments with cultured hippocampal (normally VGluT1+ and facilitating or thalamic (normally VGluT2+ and depressing) neurons demonstrated that exchanging which VGluT isoform the neurons expressed switched the direction of short-term plasticity occurring at their synapses. In other words, VGluT1-expressing synapses displayed short-term facilitation, while VGluT2- or 3-expressing synapses displayed short-term depression, regardless of the normal phenotype of the neuron (Weston et al. 2011). In addition, VGluT1 expression levels have been shown to effect vesicle pool size and quantal content (Wojcik et al. 2004; Tordera et al. 2007). However, only minor synaptic changes were observed in hippocampal slice recordings from VGluT1^+/−^ mice. VGluT1 haploinsufficiency did not appear to change hippocampal vesicular P_R_ or short-term facilitation, but seemed to reduce long-term potentiation (Fremeau et al. 2004; Balschun et al. 2010). The latter effect, however, appears to be of minor functional significance since spatial learning and memory are normal (Tordera et al. 2007; Balschun et al. 2010). Thus, a correlation between neuronal changes and behavioral deficits in VGluT1^+/−^ mice remains to be established.

One confound to the previous studies is that hippocampal neurons retain some level of VGluT2 expression, which may compensate for the VGluT1 deficiency (Weston et al. 2011). To address this issue, we have switched focus from the hippocampus to the thalamus. By modulating the excitability of thalamic relay cells, corticothalamic (CT) feedback signals can alter the likelihood that signals from sensory pathways will be transferred to the cortex (Briggs and Usrey 2008; Sherman and Guillery 2011; Crandall et al. 2015; Denman and Contreras 2015). Thus, the CT pathway has been proposed to provide the dynamic gain regulation (Ahlsén et al. 1985) underlying early attentional processing (Briggs and Usrey 2008; McAlonan et al. 2008; Saalmann and Kastner 2009).

In the present study, we show that during a sustained visual-spatial attention task, VGluT1^+/−^ mice have impaired response inhibition and decreased visual processing speed. We also demonstrate that VGluT1 haploinsufficiency increased vesicular P_R_ and reduced facilitation at CT synapses with dorsal lateral geniculate nucleus (dLGN) relay cells. These changes would dramatically limit the dynamic range for CT gain regulation of sensory throughput and could be relevant for the identified deficit in visual processing speed. Since the mouse line used in this study produces global VGluT1 haploinsufficiency, similar changes in other VGluT1-expressing synapses (ex. prefrontal connections to mediodorsal thalamus) could contribute to the attention deficit and underlie other behavioral impairments (ex. response inhibition) in VGluT1^+/−^ mice.

## Materials and Methods

### Animals

Animal procedures were approved by the Linköping ethical committee for the use of animals in research and comply with national legislation and the European Communities Council Directives of 24 November 1986 (86/609/EEC).

Eighty five mice of the VGLUT1-KO strain (*B6.129X1-Slc17a7tm1Edw/Mmcd*; MMRRC 032097-UCD-RESUS) were used in this study. This is a global VGluT1-KO strain; however, conditional VGluT1-KO mutants were not available at the time of this study. All mice were genotyped using ear clippings taken prior to weaning; behavioral mice were also verified using postmortem tail clippings. Standard PCR techniques were used (primers: WT-fwd CCAAGCAAGGTTAAGCCTAG, WT-rev GGTGAATTTGGAAAAGAGC, KO-fwd GACTCGGATCTGCATCTGCT, and KO-rev GGGGAACTTCCTGACTAGGG). Homozygous mutants were not used in this study because they are functionally blind (Johnson et al. 2007) and do not survive past weaning (Wojcik et al. 2004) without supplementary care (Herring et al. 2015). Hemizygous mutants (VGluT1^+/−^) in contrast have intact visual discrimination (Granseth et al. 2015) and survive as well as wild type (WT).

Three mice in which the tdTomato reporter was driven by the Ntsr1-Cre (*B6.Cg-Gt (ROSA)26Sortm14(CAG-tdTomato)Hze/J* strain crossed with *B6.FVB (Cg)-Tg (Ntsr1-cre)GN220Gsat/Mmcd* strain) line were used to assess colocalization between CT terminals and VGluT1 and 2 antibodies. Primers used to screen for tdTomato were: AAGGGAGCTGCAGTGGAGTA (WT-fwd), CCGAAAATCTGTGGGAAGTC (WT-rev), CTGTTCCTGTACGGCATGG (mutant-fwd), and GGCATTAAAGCAGCGTATCC (mutant-rev). Ntsr1-Cre primers were: GACGGCACGCCCCCCTTA (Ntsr1Cre-fwd) and CGGCAAACGGACAGAAGCATT (Ntsr1Cre-rev).

### Housing Conditions

For electrophysiological experiments, mice were group housed (max. four mice per cage) in passively ventilated cages (Makrolon type 2L with filter tops) or individually ventilated cages (NexGen Easy IVC) with free access to water and fed standard rodent chow. A 12:12 light:dark cycle was maintained.

For behavioral experiments, mice were single housed in passively ventilated (Makrolon type 2L without filter tops) cages with free access to water and fed standard rodent chow. Three weeks before pretraining, mice began food restriction to achieve 85 ± 5% of their free-feeding body weight. During this time, mice were familiarized with the reward (concentrated raspberry juice) in their home cages (minimum 4×). A 12:12 light:dark cycle was maintained. Each training session occurred at the same time (±1 h) during the light phase, 7 days a week.

### Immunohistochemistry and Fluorescence Microscopy

To prepare tissue for immunohistochemistry, mice were euthanized by CO_2_ inhalation and transcardially perfused with phosphate-buffered saline (PBS) followed by fixative (4% paraformaldehyde in PBS). Brains were removed immediately and postfixed for 1–3 h, then washed, and stored at 4 °C in PBS with sodium azide until sectioning. Sagittal sections, 50 μm, containing the dLGN were cut using a VT1200 vibratome (Leica Microsystems). Sections were mounted on glass coverslips and placed in 80 °C tris-EDTA (Ethylenediaminetetraacetic acid) at pH 9 for 2 min for antigenic retrieval, followed by immediate incubation in 4–8 °C tris-buffered saline (TBS; BCB-20023, Nordic Biosite). Samples were permeabilized with TBS-tween in room temperature for 1 h. Sections were incubated with primary antibody against either VGluT1 1:500 (rabbit polyclonal #135303, Synaptic Systems) or VGluT2 1:250 (rabbit polyclonal #135304, Synaptic Systems) at 4°C overnight. Samples were washed three times in TBS and incubated with secondary antibody conjugated with Alexa 488 1:200 (goat antirabbit #A11008, Life Technologies) for 1 h at 4 °C. Coverslips were washed and mounted with Vectashield (Vector Labs).

Image stacks (16-bit depth) were captured with a Zeiss 700B laser scanning confocal microscope using a 40× oil objective (NA 1.3) with 488- and 555-nm lasers (Carl Zeiss). Fluorescence channels for double staining (tdTomato and VGluT1/2) were acquired sequentially to minimize bleed-through using the same imaging parameters.

### VGluT and tdTomato Colocalization Analysis

Prior to analysis, image stacks were deconvolved using Huygens Professional software (www.svi.nl) with settings obtained from the confocal microscope. For deconvolution, the Classic Maximum Likelihood Estimation (CMLE) algorithm was applied using a theoretical point spread function, signal-to-noise ratio set to 20 for both channels and a maximum of 40 iterations with a 0.1% threshold quality change.

Subsequently, Fiji software (Schindelin et al. 2012) was used to process images and analyze tdTomato and VGluT colocalization. To identify VGluT-expressing synaptic profiles, VGluT images (488-nm channel) were first background subtracted (50 pixels rolling ball radius) and then converted to binary images. All particles smaller than one pixel were removed (erode) then a border, one pixel wide, was added to all remaining punctae (dilate). Fluorescent punctae were detected and used to calculate VGluT-profile area (μm^2^) and number of profiles per μm^2^ for each image. For sections from mice expressing tdTomato in CT neurons, a profile mask was generated from the detected VGluT-expressing punctae and overlaid on the corresponding tdTomato image (intensity range 0–65 536 AU possible for each pixel). Values for tdTomato intensity within the mask were then averaged. The mean value from each image provided an estimate of tdTomato colocalization within terminals expressing either VGluT1 or VGluT2. To provide a similar estimate of tdTomato colocalization expected from random overlap with VGluT profiles, each VGluT-profile mask was rotated 90°, 180°, and 270° on the corresponding tdTomato image. The mean intensity of tdTomato fluorescence within the mask was calculated for each rotation, and intensity values for all three rotations were averaged for each image.

### Immunogold Staining and Electron Microscopy

To prepare tissue for immunogold staining, mice were euthanized with sodium pentobarbital (200 mg/kg i.p.) and transcardially perfused, using a syringe, with PBS followed by fixative (4% paraformaldehyde—1% glutaraldehyde in PBS). Brains were dissected from the skull immediately, postfixed for 1–3 h, and then washed. The washed brain was blocked, and the dLGN containing portion was stored at 4 **°**C in PBS with sodium azide until processing.

Coronal sections (250 μm thick) were cut through the dLGN on a Vibratome. Sections encompassing the dLGN were embedded in Durcupan ACM resin or Lowicryl HM20 resin (both from Electron Microscopy Sciences) as described (Larsson et al. 2001). For morphological analysis, Durcupan-embedded tissue was cut into 40-nm sections and counterstained using uranyl acetate and lead citrate prior to examination in a JEOL 1230 electron microscope.

Ultrathin sections (70 nm) of Lowicryl-embedded tissue were subject to VGluT1 postembedding immunogold labeling as follows. First, sections were incubated in tris-buffered saline (5 mM, pH 7.4, 0.3% NaCl) with 0.1% triton X-100 (TBST) and 50 mM glycine to remove free aldehyde groups. After rinsing in TBST and blocking in TBST with 2% human serum albumin (TBST–HSA), sections were incubated in rabbit anti-VGluT1 (1:1000; Synaptic Systems; cat. 135 003) in TBST–HSA at 4°C overnight. After rinsing in TBST and incubation in TBST–HSA, sections were incubated in goat F (ab)_2_ antirabbit conjugated to 10-nm gold (British Biocell; EM.GFAR10) diluted 1:20 in TBST–HSA for 2 h. After rinsing in H_2_O, sections were counterstained with uranyl acetate and lead citrate. All sections were incubated in parallel on a Hiraoka staining plate in order to minimize labeling variability between sections.

For morphological analysis, Durcupan sections of the dLGN from three VGluT1^+/−^ and two WT mice were scanned for presumed CT terminals. These were identified based on their small size, the presence of tightly packed round vesicles, and a single asymmetric synapse. Micrographs were obtained at 100 000× magnification (pixel width 0.49 nm). Using ImageJ and the plugin PointDensitySyn ([Larsson et al. 2015]; available at https://old.liu.se/medfak/forskning/larsson-max/software), the terminal plasma membrane was outlined, the centers of all discernible synaptic vesicles marked, and the active zone (defined as the portion of presynaptic plasma membrane directly apposing the postsynaptic density) delineated. The resulting coordinate files were submitted to the second component of PointDensitySyn for computation of various measures. For analysis of VGluT1 immunogold labeling, immunolabeled Lowicryl dLGN sections from two mice of each genotype were scanned for presumed CT terminals, identified by morphology as above and by the presence of VGluT1 immunogold labeling. Immunogold labeling was analyzed in a manner similar to the synaptic vesicles, as described above. All image analysis was performed blind with respect to genotype.

### Slice Electrophysiology

For electrophysiological experiments, 6–8-week-old male or female mice (*n* = 36; 18 VGluT1^+/−^, 18 WT) were used, yielding recordings from 27 VGluT1^+/−^ relay cells and 22 WT relay cells.

To prepare dLGN slices, mice were anesthetized using isoflurane and then decapitated. The brain was rapidly removed from the skull into ice-cold artificial cerebral spinal fluid (ACSF) containing (mM) 1.25 NaH_2_PO_4_, 124 NaCl, 3 KCl, 26 NaHCO_3_, 2 MgCl_2_, 2 CaCl_2_, 3 myo-inositol, 0.5 ascorbic acid, 4 lactic acid, 10 glucose, and oxygenated with 95% O_2_, 5% CO_2_. The hemispheres were separated along the midline, and a small wedge was removed from the medial surfaces (as described in [Turner and Salt 1998]). Each hemisphere was affixed to the stage by the medial surface and sliced at a thickness of 250 μm using a Leica VT1200 vibratome. Slicing solution was ice-cold, oxygenated modified ACSF (in mM: 1.25 NaH_2_PO_4_, 6 MgCl_2_, 3 KCl, 26 NaHCO_3_, 0.5 CaCl_2_, 3 myo-inositol, 0.5 ascorbic acid, 4 lactic acid, 10 glucose, 248 sucrose). Slices containing the dLGN were transferred to oxygenated storage solution (in mM: 1.25 NaH_2_PO_4_, 124 NaCl, 3 KCl, 26 NaHCO_3_, 2 MgCl_2_, 4 CaCl_2_, 3 myo-inositol, 0.5 ascorbic acid, 4 lactic acid, 10 glucose) initially at 32–35°C and then cooled to room temperature (ca. 28°C). After a minimum recovery period of 2 h, individual slices were transferred to the patch-clamp recording chamber warmed to 35°C.

The extracellular recording solution consisted of (mM): 1.25 NaH_2_PO_4_, 124 NaCl, 3 KCl, 26 NaHCO_3_, 2 MgCl_2_, 2 CaCl_2_, 10 glucose bubbled with 95% O_2_, 5% CO_2_. 100 μM of picrotoxin, 100 μM of DL-AP5, and 200 nM of LY341495 were added to block GABA (γ-Aminobutyric acid), NMDA (N-Methyl-D-aspartic acid), and metabotropic-glutamate receptors. During experiments measuring asynchronous excitatory postsynaptic currents (EPSCs), 2 mM of CaCl_2_ was replaced with 2 mM of SrCl_2_. Cesium-based intracellular solution was used to minimize potassium currents (in mM: 100 Cs-gluconate, 10 NaCl, 10 HEPES (4-(2-Hydroxyethyl)piperazine-1-ethanesulfonic acid), 20 Triethylamine-Cl, 0.1 EGTA (Ethylene glycol-bis(2-aminoethylether)-*N,N,N,N*-tetraacetic acid), 1 Mg-ATP). About, 5 mM of QX-314 was included to block action potentials in the recorded relay cell and tracing dyes (3.25 mM of neurobiotin or 50 μM of Alexa 568) were included for postrecording visualization. A Zeiss Axioskop microscope with infrared differential interference contrast optics coupled to an ORCA-R^2^ camera (Hamamatsu) was used to visualize cells for patch-clamp recordings.

Patch electrodes were pulled from borosilicate glass capillaries (1.5/1.12 mm OD/ID, with filament; TW150F-4, World Precision Instruments) to have a resistance of 5–8 MΩ. Whole-cell voltage-clamp recordings of dLGN relay cells were acquired using a Heka EPC 9 amplifier with Pulse software or an Axon Multiclamp 700B amplifier with Clampex 10 software (Sr^2+^ experiments only). Signals were filtered at 10 kHz. The estimated junction potential (−8 mV) was compensated for at the time of recording.

Bipolar stimulating electrodes (twisted Teflon-insulated silver wires) were used to differentially stimulate optic tract (OT) or CT input by placement ventral or rostral to the dLGN, respectively (as in Fig. 2*d*). An STG 4002 stimulator (Multichannel Systems) produced charge-imbalanced biphasic pulses of 500-μs duration. The frequency of pulse delivery was controlled via a Master-8 Pulse Generator linked to the acquisition software. Stimulus amplitude (intensity) was adjusted using MC Stimulus II software. Most of the electrophysiological data were analyzed using SpAcAn and additional customized protocols written for Igor Pro version 6.22, but Clampfit version 10.7 was used to analyze asynchronous EPSCs.

### The Behavioral Chamber

Behavioral training was performed using a Bussey–Saksida Touchscreen Chamber (Campden Instruments). Each chamber was housed inside a nonilluminated sound-attenuating box. The front wall of the chamber was a touchscreen monitor covered by a black plastic mask with five horizontally aligned response windows, limiting the response area to the regions where the visual stimuli were displayed. Chambers were trapezoidal with three black plastic walls and a reward magazine accessed via a window in the wall opposite the touchscreen (in cm: 20 high × 18 long × 24 wide [at screen] or 6 wide [at magazine]). Juice reward (7 μL, unless noted otherwise) was delivered to the magazine using a peristaltic pump. The top of the chamber was covered with a transparent plastic lid. The floor was a perforated stainless steel, raised above a tray lined with wood chips.

### Five-Choice Serial Response Time Task Training and Testing

The five-choice serial response time task (5CSRTT) was run in two cohorts. Cohort 1 consisted of 23 mice (11 VGluT1^+/−^, 12 WT) that were 29–32 weeks old (mean: 30.0 ± 0.25 VGluT1^+/−^; 30.1 ± 0.32 WT) at the start of 5CSRTT training. These mice had previous experience with a two-choice compound visual discrimination task. Cohort 2 consisted of 15 male mice (8 VGluT1^+/−^, 7 WT) that were 9–12 weeks old (mean: 10.5 ± 0.46 VGluT1^+/−^; 10.8 ± 0.41 WT) and naive at the start of 5CSRTT training.

For Cohort 2, five stages of pretraining (habituation, initial touch, must touch, must initiate, and punish incorrect) preceded 5CSRTT. During “Habituation,” each mouse was placed in the behavioral chamber for 20 min (Day 1) or 30 min (Day 2) and allowed to freely explore. Approximately, 1 mL of juice was in the reward magazine at the start of the session and small volumes of the juice were delivered periodically.

“Initial touch training” consisted of two sessions of 30 min each. During these trials, the stimulus (illuminating one window of the 5CSRTT mask) was presented for 30 s. After stimulus removal, juice reward was delivered accompanied by reward tone (3 kHz) and reward tray illumination. Juice retrieval initiated an intertrial interval (ITI) of 5 s. The location of the stimulus was alternated pseudorandomly such that it never appeared in the same location more than three times in a row. If the mouse touched the displayed stimulus, an immediate triple reward (21 μL) was delivered accompanied by reward tone, reward tray illumination, and stimulus removal.

“Must touch training” (two sessions of 30 min) proceeded as in initial touch training, except that the mouse was required to touch the correct window (containing the stimulus) to trigger reward delivery. The stimulus was displayed until touched. “Must initiate training” (minimum of two sessions of 30 min, completion criterion of >30 trials in a single session) added the constraint that the mouse must initiate each new trial. Following the ITI, the reward tray was illuminated (without juice delivery) and a subsequent nose poke to the reward tray initiated display of the next stimulus and a click cue. *“*Punish incorrect training” added the constraint that touches to any incorrect windows resulted in a time-out. Time-outs were indicated by removal of the stimulus and illumination of the house LED for 10 s, followed by the standard ITI of 5 s. Training sessions (max. 30 min) continued until criterion (>30 trials per session for two sessions) was reached.

The pretraining for mice in Cohort 1 was similar except that the touchscreen mask only had two windows, as these mice were being prepared for a two-choice visual discrimination. After completion of the two-choice visual discrimination testing, mice from Cohort 1 were provided with one session of must initiate training with the 5CSRTT mask and we found that they easily transferred to this new task, responding to the correct window with a latency of approximately 4 s. Thus, they moved directly to 5CSRTT training Stage 3 in the following session.

**Figure 1:**
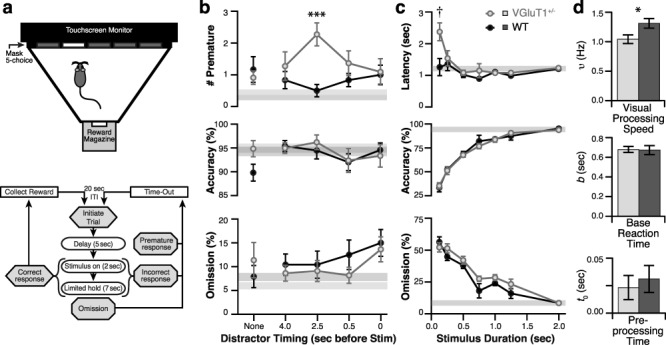
The 5CSRTT reveals deficits in visual attention and inhibitory response control in VGluT1^+/−^ mice. (*a*) Schematic illustration of the 5CSRTT behavioral chamber (top) and behavioral protocol (bottom). See Methods section for detailed description. (*b*) Performance during the distractor test. Graphs plot premature responses (top), accuracy (middle), and omissions (bottom) versus the different distractor conditions tested. Data from 11 VGluT1^+/−^ and 12 WT mice plotted as group means. Error bars show SEM, omitted when less than the diameter of the symbol. Gray bars show baseline performance for WT (dark) and VGluT1^+/−^ (light). Sidak’s multiple comparison test: ^*^^*^^*^, *P* = 0.0004. (*c*) Performance during the stimulus duration test. Graphs plot correct response latency (top), accuracy (middle), and omissions (bottom) versus the different stimulus durations tested (*n* = 16 VGluT1^+/−^ and 15 WT mice), plotted as in (*b*). Since baseline performance was similar, these bars are almost entirely overlapping. Sidak’s multiple comparison test: **†**, *P* < 0.0001. (*d*) Parameters of visual processing acquired through TVA analysis of individual mice during the stimulus duration test. Preprocessing time (*t_0_*) and base reaction time (*b*) were not different between genotypes. Visual processing speed (*υ*) was significantly slower in VGluT1^+/−^ mice (two-tailed *t*-test: ^*^, *P* = 0.02).

5CSRTT training trials followed the protocol outlined in Figure 1*a*. Trials were initiated with a nose poke to the reward tray, followed by a 5-s delay before the stimulus was displayed in one of the five touchscreen windows. If the mouse touched any window during the delay, it was scored as a “premature response” and resulted in a time-out followed by a repeat of the same trial. Stimuli were displayed for a set duration (stimulus duration). Touches to the window containing the stimulus were scored as “correct” (*C*, resulting in reward), while touches to any other window were scored as “incorrect” (*I*, resulting in a time-out). If the mouse failed to respond to any window during stimulus display, the trial entered a limited hold period during which correct or incorrect touches were still applicable. If the mouse still failed to respond to any window during the limited hold, it was scored as an “omission” (*O*) and resulted in a time-out followed by advancement to the next trial. A 20-s ITI followed each reward collection or time-out before the next trial could be initiated. Each session was limited to a maximum of 60 trials or 40 min. 5CSRTT training progressed through four stages during which the stimulus duration and limited hold were reduced as outlined in Table 1.

**Table 1:** Outline of the four training stages of 5CSRTT

	Stimulus duration (s)	Limited hold (s)	Criterion (three of four consecutive sessions)
Stage 1	16	21	>35 trials > 80% accuracy; < 25% omissions
Stage 2	8	13	
Stage 3	4	9	≥50 trials > 80% accuracy; < 20% omissions
Stage 4	2	7	

Performance during each session was assessed with two measures: % accuracy = *C/(C + I)* and % omission = *O/(O + C + I)*. While premature responses were not directly restricted, we found that high levels of premature responding interfered with the ability to meet the minimum trial criterion. After passing criterion on Stage 4 of 5CSRTT training, each mouse performed 2–3 additional sessions with stimulus duration fixed at 2 s and a maximum number of trails limited to 50 (no max time) to establish baseline performance.

### Attention Testing with 5CSRTT

Test sessions (TS) and interpolated baseline sessions were limited to 50 trials with no time limit. This assured that all mice completed the minimum number of trials for each test variable. The basic protocol during testing was identical to that used for the baseline 5CSRTT except for the attention-challenging parameters discussed below.

During the “stimulus duration test” protocol, the stimulus duration was pseudorandomly varied across trials. Different stimulus durations were tested across one or two pairs of TS separated by a minimum of 9 days (interpolated baseline testing). The tested durations (in s) were as follows for Cohort 1: 2.0, 1.0, 0.5, 0.25, and 0.125 durations each having 10 occurrences (two per window location) per session (TS1–2). Thus, each stimulus duration was repeated 20 times with a balanced location distribution. Tested durations for Cohort 2 were (in s): 2.0, 1.25, 1.0, 0.75, and 0.5 s duration (TS1–2) or 2.0, 1.0, 0.5, 0.25, and 0.125 s durations (TS3–4) each having 10 occurrences (two per window location) per session. Thus, stimulus durations of 2.0, 1.0, and 0.5 s were repeated 40 times (location balanced) while those of 1.25, 0.75, 0.25, and 0.125 s were only repeated 20 times (location balanced). In addition to evaluating the standard response parameters, we applied a recently developed mathematical model based on Bundesen’s theory of visual attention (TVA) to estimate visual processing speeds and other parameters of attentional capacity. Using the formulae provided by (Habekost 2015), we calculated three parameters for each mouse by applying simultaneous least-sum-of-squares optimized curve fits to “mean score” and “correct response latency” versus stimulus duration using Igor Pro. Mean score was calculated as *C/(C + I + O)*. The first parameter evaluates the preprocessing time (*t_0_*), the time needed to orient toward and compute attentional weights for the presented stimulus. The second, visual processing speed (*υ*), is a rate parameter associated with sampling and encoding the target stimulus. The third parameter, base reaction time (*b*), reflects the time required to execute the motor response. The parameters *υ* and *b* reflect purely perceptual and purely motor processes, respectively, while *t_0_* is a blending of perceptual and motor processes.

During the “distractor test,” the stimulus duration remained at the training value (2 s) and a distracting noise (500 ms white noise at 80–90 dB) was played during the 5-s delay period. This distractor could occur simultaneously with the onset of the stimulus, or 0.5, 2.5, or 4 s before stimulus onset. Furthermore, on 20% of the trials, the distractor was omitted to reduce within session habituation. Distractor timing was pseudorandomly varied across trials such that 10 repeats of each condition were distributed throughout each TS in a location-balanced manner. To reduce between session habituation to the distractor, the two distractor TSs were separated by one baseline 5CSRTT session.

### Statistical Analysis

Data are reported as mean ± standard error of mean (SEM); except for the electron microscopy (EM) density values, which were not normally distributed and are thus reported as median ± interquartile range. Statistical comparisons were performed using GraphPad Prism 7 software. Statistical tests were unpaired *t*-test, unless mentioned in the text and/or figure legend. *P* values less than 0.05 were considered significant.

## Results

### Visual Attention is Impaired in Mice with VGluT1 Deficiency

To determine if mice with VGluT1 deficiency have difficulties with visual attention, we compared the performance of VGluT1^+/−^ mice and littermate controls (WT) during the 5CSRTT. The 5CSRTT is a well-established method for evaluating sustained visual attention in rodents (Bari et al. 2008). The protocol for a standard trial of the 5CSRTT is outlined in the flow chart in Figure 1*a* and described in detail in the Methods section. For our experiments, we chose to evaluate attention using two methods: making the visual stimulus more difficult to detect (reducing stimulus duration from baseline of 2 s) or adding a distraction (a loud white-noise sound played during the delay).

During the distractor test, we evaluated the performance of 23 mice (Fig. 1*b*; 11 VGluT1^+/−^ and 12 WT from Cohort 1 only) across two TS (separated by one baseline session to reduce habituation to the distractor). During TS, a distractor (500-ms white noise at 80–90 dB) was sounded during the delay period (0, 0.5, 2.5, or 4 s before stimulus onset), except for 20% of the trials when the distractor was omitted to serve as an internal control. All mice met training criteria during internal control trials. VGluT1^+/−^ mice showed a significant increase in the likelihood of making a premature response when the distractor sounded 2.5 s before stimulus onset (VGluT1^+/−^ versus WT *P* = 0.0004, Sidak’s multiple comparisons following two-way ANOVA with interaction *P* = 0.011). This 4-fold increase in premature responding likely reflects a breakdown in inhibitory response control. The distractor had no effect on accuracy (interaction *P* = 0.385, distractor timing *P* = 0.145, genotype *P* = 0.439; two-way Analysis Of Variance (ANOVA)); and while the timing of the distractor did influence omissions, the increase was small (within training criterion) and was similar for both genotypes (interaction *P* = 0.643, distractor timing *P* = 0.041, genotype *P* = 0.538; two-way ANOVA). Thus, the distractor did not significantly disrupt the allocation of spatial attention.

During the stimulus duration test, we evaluated the performance of 38 mice (Fig. 1*c*; 19 VGluT1^+/−^ and WT, in two cohorts) across 2–4 TSs. During each TS, the stimulus duration (2.0, *1.25*, 1.0, *0.75*, 0.5, 0.25, or 0.125 s; *italicized* values only tested in Cohort 2) was pseudorandomly varied across trials and all mice that failed to meet accuracy or omission training criteria during the 20% of test trials having stimulus duration of 2.0 s were removed from the analysis (three VGluT1^+/−^ and four WT). No effects on premature responding were observed (data not shown). Both genotypes showed a decrease in accuracy and increase in omissions as stimulus duration decreased from the baseline value of 2.0 s. No genotype differences in accuracy were observed (interaction *P* = 0.98, stimulus duration *P* < 0.0001, genotype *P* = 0.789; two-way ANOVA), indicating similar levels of engagement and retention of the task. Omissions were significantly higher in the VGluT1^+/−^ group (interaction *P* = 0.348, stimulus duration *P* < 0.0001, genotype *P* = 0.0297; two-way ANOVA), indicating a deficit in attention relative to WT. When stimulus duration was reduced to 0.125 s, the task became too difficult for either genotype to reliably detect the stimulus thus a ceiling effect was observed. Interestingly, VGluT1^+/−^ mice were more likely to guess when the stimulus was so brief, as indicated by the slightly lower omissions and significant increase in correct response latency (0.125 s VGluT1^+/−^ versus WT *P* < 0.0001, Sidak’s multiple comparison following two-way ANOVA with interaction *P* = 0.003).

In order to relate these results to specific cognitive functions, we applied the TVA model (Fitzpatrick et al. 2017). From this model, we derived three basic parameters: *visual processing speed* (perceptual), *preprocessing time* (motor and perceptual), and *base reaction time* (motor). Our results showed that visual processing speed was significantly reduced in VGluT1^+/−^ mice (1.043 ± 0.074 Hz) compared to WT (1.311 ± 0.081 Hz; *P* = 0.020), while preprocessing time (*P* = 0.634) and base reaction time (*P* = 0.899) were not affected by genotype (Fig. 1*d*). Reduced visual processing speeds have previously been demonstrated in patients with Attention Deficit Hyperactivity Disorder (Habekost 2015) and in rodents treated with scopolamine (Fitzpatrick et al. 2017). Scopolamine is a muscarinic antagonist known to induce attentional deficits in the 5CSRTT (de Bruin et al. 2006). Thus, the reduction in visual processing speed that we observed suggests that VGluT1 deficiency impairs the enhanced sensory processing that is normally produced by attention (McAlonan et al. 2008).

### Basic Synaptic Properties are Unaffected by VGluT1 Deficiency

**Figure 2:**
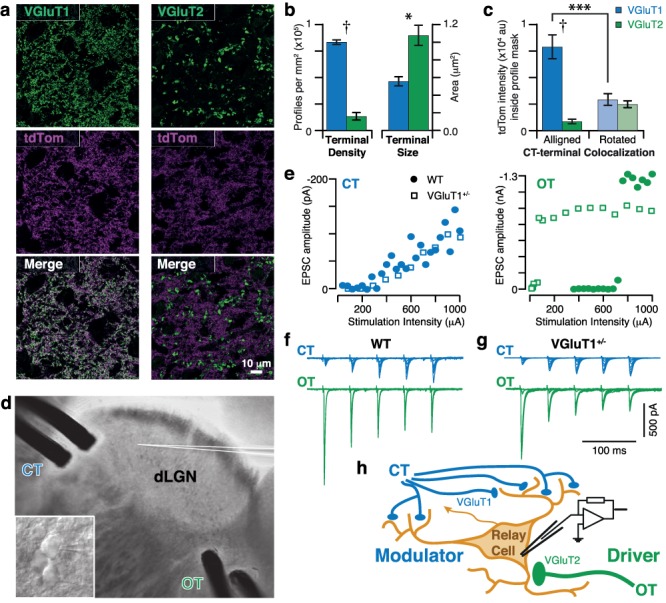
Dichotomy in VGluT expression and basic response properties of OT and CT inputs to dLGN. (*a*) Representative confocal images of immunostained dLGN sections from an Ntsr1-tdTomato mouse. VGluT1 (left) or VGluT2 (right) immunostaining is shown in green. CT terminals in the dLGN are labeled with tdTomato (magenta). Merged images reveal colocalization (white). (*b*) Summary of terminal size and density for VGluT1+ (blue) versus VGluT2+ (green) profiles from three Ntsr1-tdTomato mice. Mean ± SEM. Two-tailed *t*-test values: ^*^, *P* = 0.014; **†**, *P* < 0.0001. (*c*) Quantification of colocalization between tdTomato and VGluT1+ (blue) versus VGluT2+ (green) profiles from three Ntsr1-tdTomato mice. For each confocal image, a mask was created from the immunostained channel. The total intensity of tdTomato fluorescence within this mask was quantified (left). TdTomato intensity corresponding to random colocalization (rotated) is plotted to the right for both VGluT1+ and VGluT2+ profiles. Mean ± SEM. RM one-way ANOVA *P* < 0.0001. Sidak’s multiple comparison values: ^*^^*^^*^, *P* = 0.0003; **†**, *P* < 0.0001. (*d*) Image of dLGN slice preparation showing positioning of recording and stimulating electrodes. CT inputs were stimulated with a bipolar electrode at the rostral margin of the dLGN. Retinal inputs were stimulated with an electrode where OT fibers cross into the dLGN. A patch-clamp electrode (white lines) was used to record from a dLGN relay cell (magnified, inset). (*e*) Sample recruitment curves following stimulation of CT (blue, left) or OT (green, right) inputs to dLGN relay cells in WT (solid symbols) or VGluT1^+/−^ (open symbols) mice. EPSC amplitudes from EPSC_1_ of five-pulse trains (shown in *f* and *g*) during incremented stimulus intensity. (*f*) Example traces recorded from a WT relay cell. Multiple traces with increasing stimulus intensity are overlaid. Stimulus trains of five pulses (20 Hz) illustrate the characteristic patterns of facilitation or depression observed upon stimulation of CT or OT inputs, respectively. (*g*) Same as in *f* performed in slices from a VGluT1^+/−^ mouse. Illustrating that CT input continued to produce facilitating responses and OT inputs remained depressing in these mice. (*h*) Illustration of the dichotomy between CT and OT inputs to relay cells. CT inputs to relay cells are quintessential modulator*-*type synapses, while OT inputs are driver-type synapses. Anatomical data suggests that CT and OT inputs rely on VGluT1 (blue) and VGluT2 (green), respectively.

To determine the mechanism that makes attentive modulation inefficient in VGluT1^+/−^ mice, we investigated the synaptic properties of CT inputs to relay cells in the dLGN (the thalamic nucleus via which sensory information from the retina is relayed to the primary visual cortex). We chose to focus on this synapse for two reasons. First, CT feedback was shown to modulate relay cell gain, and this modulation of sensory throughput has been proposed as a mechanism for attentional processing (Ahlsén et al. 1985; Briggs and Usrey 2008; Crandall et al. 2015; Denman and Contreras 2015). Second, the synapses, which transfer sensory signals from the retina to cortex (OT to relay cell to cortical layer 4), are of the driver type and typically express VGluT2; while modulatory CT synapses express VGluT1 (Fig. 2*h*) (Fujiyama et al. 2003; Land et al. 2004; Nakamura et al. 2007; Yoshida et al. 2009; Sherman and Guillery 2011). Thus, VGluT1 deficiency would be expected to impact CT feedback while leaving incoming visual signals intact.

We used the Ntsr1-Cre (GN220) mouse line to confirm that CT synapses selectively use VGluT1 and not VGluT2. The Ntsr1-Cre (GN220) mouse has been shown to target CT neurons with high specificity (Bortone et al. 2014; Kim et al. 2014; Sundberg et al. 2018). When crossed with a tdTomato reporter mouse line, bright fluorescence was observed throughout the cytosol of the CT neuron, including the axon terminals in the thalamus (magenta, Fig. 2*a*). This labeling allowed us to examine the degree of colocalization between CT terminals and immunofluorescence for VGluT1 or 2. As expected, confocal images revealed distinct staining patterns for VGluT1 and 2 (green, Fig. 2*a*), which correspond to the expected structure of CT and OT terminals, respectively. VGluT1+ profiles were small (area: 0.56 ± 0.05 μm^2^) and numerous (83.4 ± 2.1 thousand profiles per mm^2^), while VGluT2+ profiles were larger (area: 1.08 ± 0.11 μm^2^) and less numerous (13.4 ± 3.6 thousand profiles per mm^2^) (Fig. 2*b*). Qualitative examination of the merged images of tdTomato and VGluT1-immunostaining revealed a high degree of colocalization. In contrast, sections stained for VGluT2 showed almost no colocalization with tdTomato (white, Fig. 2*a*).

To quantify the colocalization between the CT terminals and the VGluT-profiles, we first converted the individual VGluT images into binary images (see Methods section), then each VGluT profile (green channel) was used as a mask to examine the corresponding tdTomato fluorescence image (magenta channel). For each section (*n* = 6; 3 mice × 2 antibody stains), the mean intensity of tdTomato fluorescence within the mask was calculated. As a control for random overlap, we also calculated the mean tdTomato intensity when the mask for each section was rotated at 90°, 180°, and 270°. The results of this analysis (Fig. 2*c*) showed that tdTomato intensity within the VGluT1-profile mask (7903 ± 1126 au) was significantly greater than expected by random overlap (2925 ± 550 au; *P* = 0.0003, Sidak’s multiple comparison following one-way RM-ANOVA). In contrast, tdTomato intensity within the VGluT2-profile mask (868 ± 213 au) was less than expected by random overlap (2485 ± 322 au), but not significantly (*P* = 0.166). The amount of colocalization between VGluT1 and tdTomato was almost a degree of magnitude (9.1-fold) greater than that between VGluT2 and tdTomato (*P* < 0.0001). This result confirms that CT terminals preferentially express VGluT1 and exclude VGluT2.

Frequently, when one isoform of a gene is knocked out, the expression patterns of other isoforms changes in a compensatory manner. Previous studies have shown that, at the whole brain level, this is unlikely for VGluT1 knockout mice (Wojcik et al. 2004). However, we wanted to be certain that there are no changes at the local circuit level. Therefore, we examined the expression patterns of VGluT1 and 2 in the dLGN of VGluT1^+/−^ mice and found that terminals expressing either isoform displayed the same size and density patterns as in WT controls (*n* = 7 sections; terminal density: interaction *P* = 0.940, genotype *P* = 0.991, VGluT type *P* < 0.0001; terminal size: interaction *P* = 0.886, genotype *P* = 0.559, VGluT type *P* < 0.0001; two-way RM-ANOVA). VGluT1+ profiles were small (area in μm^2^: 0.46 ± 0.02 WT vs. 0.41 ± 0.02 VGluT1^+/−^) and numerous (density in thousands of profiles per mm^2^: 91.0 ± 5.2 WT vs. 90.8 ± 4.5 VGluT1^+/−^) irrespective of genotype (*P* = 0.823 and 0.998, respectively; Sidak’s multiple comparison). VGluT2+ profiles were large (area in μm^2^: 1.26 ± 0.08 WT vs. 1.23 ± 0.12 VGluT1^+/−^) and less numerous (density in thousands of profiles per mm^2^: 25.6 ± 1.8 WT vs. 25.9 ± 2.2 VGluT1^+/−^) in both genotypes (*P* = 0.945 and 0.998; Sidak’s multiple comparison; Fig. S1). Thus, there is no indication of a change in isoform expression. Colocalization with CT terminals could not be assessed due to difficulties with crossbreeding the Ntsr1-Cre and VGluT1^+/−^ mouse lines.

The signaling properties of CT terminals are also distinctively different from OT terminals. CT inputs to relay cells are archetypal “modulator” type synapses, characterized by many distal synaptic contacts, small unitary responses, and short-term facilitation. In contrast, OT inputs are “driver-”type synapses, characterized by few proximal synaptic contacts, large unitary responses, and short-term depression (Sherman and Guillery 2011).

To investigate if this dichotomy was intact in VGluT1 hemizygous mice, we compared EPSCs recorded from dLGN relay cells in brain slices (Fig. 2*d*) from VGluT1^+/−^ and WT mice. We assessed three of the properties that distinguish modulatory and driving synapses (unitary response size, synapse number, and short-term synaptic plasticity) during electrical stimulation of CT or OT inputs. The stimulus was a series of five-pulse trains (20 Hz) repeated with incrementing stimulus intensities (20–30 s between trains). CT recruitment curves, for the first EPSC in each train (EPSC_1_), show that EPSC amplitude increased gradually with increments in stimulus intensity above threshold. This suggests that each relay cell receives a large number of synapses arising from many different CT axons with small unitary contributions (Turner and Salt 1998; Granseth and Lindström 2003). In contrast, OT recruitment curves increased in a step-wise manner, indicative of a low number (typically 1–2) of axons each contributing a large unitary response (Fig. 2*e*). Threshold values varied between cells, but the general pattern, gradual (CT) versus step wise (OT), was consistent across cells and genotypes. In addition, the sign of short-term synaptic plasticity observed during each stimulus train (facilitation for CT and depression for OT stimulation) was not different between WT and VGluT1^+/−^ mice (Fig. 2*f*,*g*).

In conclusion, our results support the model of dLGN input presented in Figure 2*h* and demonstrate that a general reduction in VGluT1 expression, as in VGluT1^+/−^ mice, does not alter the modulator/driver dichotomy of CT and OT synapses. However, the magnitude of facilitation produced by CT stimulation does appear to be reduced in VGluT1^+/−^ mice (Fig. 2*f*,*g*). Thus, the properties of short-term facilitation at CT synapses were examined in greater detail using paired-pulse and high-frequency train stimulation paradigms.

### VGluT1 deficiency reduces build-up and maintenance of short-term facilitation at CT synapses

**Figure 3:**
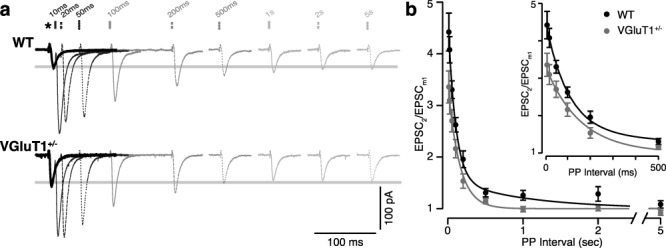
Paired-pulse stimulation reveals the absence of the slow phase of facilitation in VGluT1^+/−^ mice. (*a*) Sample EPSCs recorded from a WT (top) and VGluT1^+/−^ (bottom) relay cell during paired-pulse stimulation. CT inputs were stimulated with PPI as indicated (bars above). Current traces are averages of 3–5 repeats. Paired-pulse repeats were separated by 30 s to allow recovery from previous facilitation. Traces are aligned by the first pulse in each pair (^*^). For PPI > 500 ms, the time between EPSC_1_ and EPSC_2_ has been cropped for display purposes. The thick gray lines indicate the mean amplitude of EPSC_1_ across all PPI (EPSC_m1_) for each cell. (*b*) Mean facilitation values for each PPI are plotted for WT (*n* = 10; black dots) and VGluT1^+/−^ (*n* = 9; gray dots). Error bars are SEM. For both genotypes, facilitation decreases exponentially with increasing PPI. WT data are best fit by a double exponential curve (black), while VGluT1^+/−^ data only require a single exponent (gray) with a time course similar to the fast component of the WT data. Inset shows the shorter PPI on an expanded time scale.

Paired-pulse stimulation of CT inputs revealed a significant reduction in facilitation in VGluT1^+/−^ mice (Fig. 3*a*). CT inputs were stimulated with paired pulses separated by intervals (PPI) between 10 ms and 5 s. For each cell, the series of PPI was repeated 3–5 times with a 30-s recovery period between each pair of pulses. Recorded current traces were averaged for each PPI before measuring peak amplitudes of EPSC_1_ and EPSC_2_. Paired-pulse facilitation was calculated as the amplitude of EPSC_2_ for each PPI divided by the mean amplitude of EPSC_1_ across all PPI (EPSC_m1_). Plots of the mean facilitation values for 10 WT and 9 VGluT1^+/−^ recordings show that facilitation decayed exponentially with increasing PPI. For WT mice, these data were best fit by a double exponential curve with fast (*A* = 2.75 ± 0.35, *τ* = 99 ± 23 ms) and slow (*A* = 0.56 ± 0.33, *τ* = 1.0 ± 0.83 s) components. In contrast, the data from VGluT1^+/−^ mice were best fit with a single exponential (*A* = 2.21 ± 0.17, *τ* = 154 ± 20 ms) that was similar to the fast component of the WT data. Thus, the slow phase of facilitation appears to be absent in VGluT1^+/−^ mice (Fig. 3*b*).

CT stimulation with high-frequency trains revealed significant reductions in steady-state facilitation and earlier induction of synaptic depression in VGluT1^+/−^ mice. We stimulated CT inputs with long (20 s) trains at frequencies of 5, 10, or 20 Hz. Each frequency train was repeated 2–3 times (with 2-min recovery period between trains) and the resulting current traces averaged. Facilitation values for each frequency train were calculated as the amplitude of EPSC_n_ divided by the mean amplitude of EPSC_1_ across all frequency trains (EPSC_m1_).

**Figure 4:**
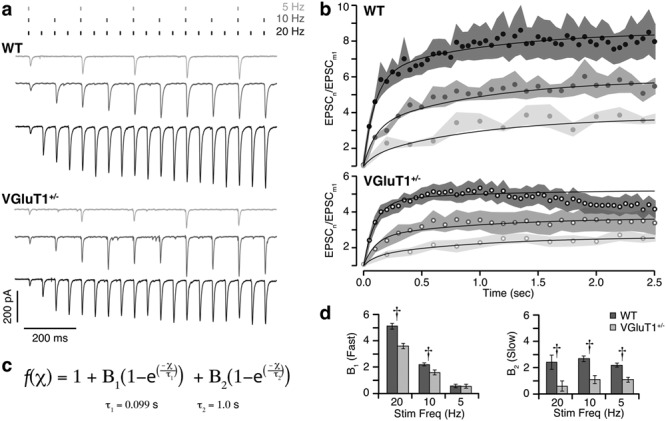
Steady-state levels of facilitation during train stimulation are reduced in VGluT1^+/−^ mice. (*a*) Example of EPSCs recorded from a WT (top) and VGluT1^+/−^ (bottom) relay cell during the first 2.5 s of a 20-s train stimulation. CT inputs were stimulated with trains of pulses at 20, 10, and 5 Hz (dashes above). Displayed current traces are averages of 2–3 repeats. Facilitation continues to build beyond the second pulse, and the steady-state level reached depends upon stimulation frequency. Steady-state facilitation is clearly reduced in the VGluT1^+/−^ cell. (*b*) Mean facilitation values are plotted versus time for WT (*n* = 4–5; filled dots) and VGluT1^+/−^ (*n* = 7–8; empty dots). Stimulation frequency is indicated in gray scale (as in *a*). Shaded regions show SEM. (*c*) Equation that was fit to facilitation values during the first 2.5 s of train stimulation, plotted as solid black curves in *b*. Values for *τ*_1_ and *τ*_2_ were measured from paired-pulse recordings in WT (Fig. 3b). (*d*) Comparisons of the magnitude (*B*) of fast (*τ*_1_, left) and slow (*τ*_2_, right) components of facilitation during 20-, 10-, and 5-Hz trains. As expected, the contribution of the fast component decreases with stimulation frequency for both genotypes. The contribution of the slow component is stable across stimulation frequencies and consistently smaller in VGluT1^+/−^ compared to WT cells. Error bars show standard deviation of the fit. Two-way ANOVA, Sidak’s multiple comparison values: **†**, *P* < 0.0001.

The buildup of facilitation during the initial portion (first 2.5 s) of each high-frequency train was clearly reduced in CT synapses of VGluT1^+/−^ mice. In WT mice, facilitation builds with subsequent stimuli until reaching a steady-state level that is dependent upon stimulation frequency (Fig. 4*a*,*b*). To compare the buildup in facilitation between genotypes, we fit each train (Fig. 4*b*) with a function describing the accumulation of two exponentially facilitating components (equation: Fig. 4*c*). We constrained the curve fitting to the time constants derived from the paired-pulse experiments (*τ*_1_ = 99 ms and *τ*_2_ = 1.0 s), as these values should not be confounded by differences in synaptic depression (discussed later). This analysis revealed a consistent reduction in the slow component of facilitation (*B*_2_) in VGluT1^+/−^ mice (20 Hz: *B*_2_ = 0.61 ± 0.40; 10 Hz: *B*_2_ = 1.10 ± 0.30; 5 Hz*: B*_2_ = 1.09 ± 0.19) relative to WT (20 Hz: *B*_2_ = 2.42 ± 0.55; 10 Hz: *B*_2_ = 2.68 ± 0.21; 5 Hz: *B*_2_ = 2.20 ± 0.16; two-way ANOVA*:* interaction *P* = 0.067, stimulation frequency *P* = 0.048, genotype *P* < 0.0001). The fast component (*B*_1_) was also significantly reduced in VGluT1^+/−^ mice (20 Hz*: B*_1_ = 3.61 ± 0.21; 10 Hz: *B*_1_ = 1.58 ± 0.19; 5 Hz*: B*_1_ = 0.54 ± 0.16) relative to WT (20 Hz*: B*_1_ = 5.11 ± 0.23; 10 Hz*: B*_1_ = 2.20 ± 0.11; 5 Hz*: B*_1_ = 0.57 ± 0.14; two-way ANOVA*:* interaction, stimulation frequency, and genotype *P* < 0.0001), but only at the 10- and 20-Hz frequencies (Fig. 4*d*).

**Figure 5:**
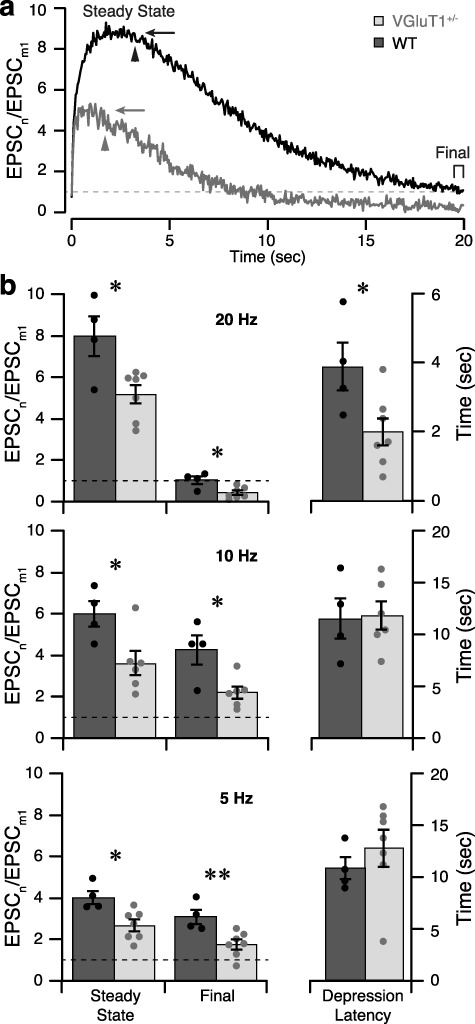
High-frequency stimulation causes earlier and more complete synaptic depression in VGluT1^+/−^ mice. (*a*) Plots of facilitation recorded from a WT (black) or VGluT1^+/−^ (gray) relay cell during 20 s of 20-Hz CT-stimulation (same cells as in Fig. 4). Steady-state facilitation (arrow), depression latency (arrowhead), and final facilitation (bracket) are indicated for each cell. The dashed line indicates facilitation of 1 (that is, an EPSC amplitude equivalent to EPSC_1_). (b) Group means for steady-state and final facilitation levels (left) and depression latency (right) during 20-Hz (top), 10-Hz (middle), and 5-Hz (bottom) train stimulation. Histograms show the mean values for WT (*n* = 4; dark) and VGluT1^+/−^ (*n* = 6–7; light) with error bars for SEM. Dots show the distribution of values from individual cells. The degree of facilitation is significantly lower in VGluT1^+/−^ cells at both time points across all stimulus frequencies. During the 20-Hz train, VGluT1^+/−^ mice have a significantly shorter depression latency than WT. The *t*-test comparisons between WT and VGluT1^+/−^, ^*^P < 0.05; ^*^^*^P < 0.01.

With continued high-frequency stimulation, additional differences between VGluT1^+/−^ and WT mice were observed. For example, during 20-Hz stimulation, synaptic depression (likely due to vesicle depletion) began to counteract facilitation relatively early (2–4 s) and eventually reduced EPSC amplitudes to a level at or below that of EPSC_m1_ (Fig. 5*a*). In the WT recording, nearly 20 s of stimulation was required to produce sufficient depression to reduce EPSC amplitudes to 1 (equivalent to the amplitude of EPSC_m1_). In comparison, the VGluT1^+/−^ recording depressed to 1 after approximately 10 s and continued to depress further during the remaining 10 s of stimulation. Thus, the final EPSC amplitude was actually smaller than the initial, unfacilitated state. To quantify these differences, three measures of long-train facilitation were calculated for each cell: (1) “steady-state facilitation” was calculated by fitting a horizontal line through the region of peak facilitation, (2) “depression latency” was defined as the time point when facilitation fell below steady-state levels for the final time, and (3) “final facilitation” was defined as the mean value of facilitation during the last 10 stimulations of each train (Fig. 5*a*).

By examining all stimulation frequencies, we found that steady-state (all frequencies *P* < 0.05) and final facilitation (20 and 10 Hz *P* < 0.05; 5 Hz *P* < 0.01) levels were significantly lower in VGluT1^+/−^ (*n* = 7) mice than in WT (*n* = 4; Fig. 5*b*). For both genotypes and at all stimulation frequencies, there was some degree of depression during the train (final facilitation was lower than steady state); however, only during 20-Hz stimulation did this depression fully overcome the initial facilitation (return to ≤1). Specifically, final facilitation during 20-Hz WT recordings was 1.03 ± 0.19, while VGluT1^+/−^ values were significantly lower (0.42 ± 0.10; *P* = 0.012). Depression latencies were similar between genotypes during 5-Hz (*P* = 0.43) and 10-Hz (*P* = 0.92) trains. However, during the 20-Hz train, VGluT1^+/−^ recordings began to depress 2 s earlier than WT (2.0 ± 0.39 s versus 3.9 ± 0.70 s; *P* = 0.030; Fig. 5*b*).

### VGluT1 Deficiency Reduces Presynaptic VGluT1 Levels, but not Synaptic Vesicle Number

During 20-Hz stimulation, we observed final facilitation values of ≤1 and a shorter depression latency at VGluT1^+/−^ synapses, suggesting that less neurotransmitter is released when CT synapses are challenged by high synaptic vesicle release rates. Tordera and colleagues showed reduced numbers of vesicles in hippocampal synapses from VGluT1^+/−^ mice, which could make synapses more prone to depression. Furthermore, western blots showed that VGluT1 protein levels were reduced by approximately 40% (without compensatory upregulation of VGluT2), which could make neurotransmitter refilling less efficient (Tordera et al. 2007). Since the findings above are from hippocampal samples, we first wanted to determine if the same changes are occurring at CT terminals in the dLGN. To do this, we examined thalamic sections from VGluT1^+/−^ or WT mice with EM and immunogold labeling for VGluT1.

**Figure 6:**
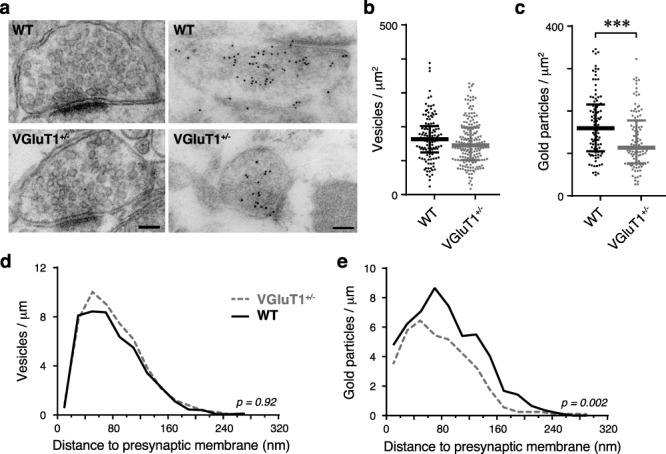
Ultrastructural analysis of synaptic vesicles and VGluT1 immunogold labeling in CT terminals of WT and VGluT1^+/−^ mice. (*a*) Electron micrographs of terminals of presumed CT origin in nonimmunolabeled (left panels) and VGluT1 immunogold-labeled (right panels) ultrathin sections through the dLGN of WT and VGluT1^+/−^ mice. Scale bars are 100 nm, valid for left and right panels, respectively. (*b,c*) Quantification of the synaptic vesicle density (*b*) and VGluT1 immunogold labeling (*c*) in CT terminals. While vesicle number is unchanged, the VGluT1 labeling is significantly reduced. Bars indicate median and interquartile range. Two-tailed Mann–Whitney *U*-test values; ^*^^*^^*^, *P* < 0.001. (*d,e*) Distribution of the perpendicular distance between vesicles (*d*) or immunogold particles (*e*) and the presynaptic plasma membrane of the active zone in CT terminals, normalized by active zone length. Only vesicles/particles that could be orthogonally projected on the active zone membrane were included. No significant difference in the distribution of vesicles was detected; however, the distribution of immunogold particles differed significantly between WT and VGluT1^+/−^ mice (Kolmogorov–Smirnov test, P values indicated in plots).

At the EM level, key morphological features can be used to distinguish between the presynaptic terminals arising from the OT or CT pathways (Sherman and Guillery 2011). VGluT1-gold particles were found to label profiles that were consistent with previous descriptions of CT terminals (Fig. 6*a*). Comparisons between VGluT1^+/−^ (3 mice, 181 terminals) and WT (3 mice, 123 terminals) samples revealed no difference in synaptic terminal size (maximum Feret diameter = WT: 677 ± 15 nm; VGluT1^+/−^: 685 ± 14 nm), synapse length (WT: 219 ± 5 nm; VGluT1^+/−^: 213 ± 4 nm), or number of synaptic vesicles per terminal (Fig. 6*b*). However, profiles in VGluT1^+/−^ mice showed a significant reduction in gold particle density (Fig. 6*c*). This difference corresponds to a 28.9% reduction of VGluT1 levels. Further analysis of the distribution of both vesicles and VGluT1-gold particles relative to the active zone revealed a significant reduction in gold particles at all distances (Fig. 6*e*; *P* = 0.002, Kolmogorov–Smirnov test), while no distance effect was evident for vesicle distribution (Fig. 6*d*; *P* = 0.92, Kolmogorov–Smirnov test).

In conclusion, the reduced facilitation and early depression at CT terminals cannot be explained by reduced vesicle numbers. Nevertheless, the almost 30% reduction in VGluT1 levels could reduce the vesicle-filling rate, which might lead to release of partially filled vesicles during high-frequency stimulation. This quantal size (*Q*) effect could explain why the VGluT1^+/−^ effects are less prevalent at lower stimulation frequencies (5 and 10 Hz), where vesicle refilling can keep up with vesicle recycling. However, vesicle recycling rates should have negligible impact on the paired-pulse protocols used in this study, so any reduction in vesicle-refilling rate would be insufficient to produce the significant decrease in paired-pulse facilitation we observed. To resolve this issue, we decided to examine *Q* and initial P_R_ at CT synapses. Since facilitation values are normalized to the amplitude of EPSC_m1_, initial differences in P_R_ and/or *Q* would affect all measures of facilitation.

### VGluT1 Deficiency Alters P_R_, but not *Q*

**Figure 7:**
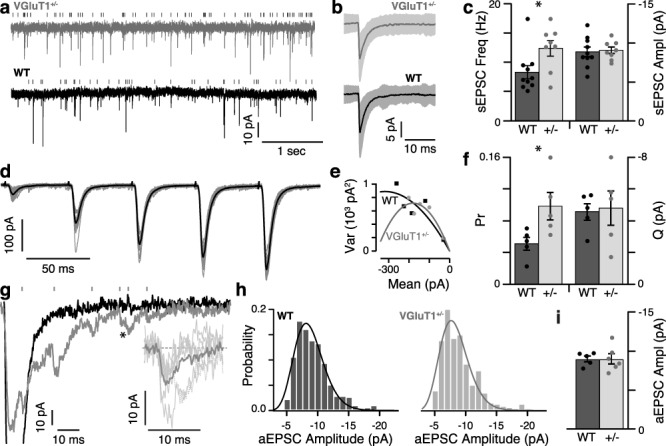
Initial P_R_ is increased in VGluT1^+/−^ mice, while *Q* is unchanged. (*a*) Example traces of sEPSCs recorded from VGluT1^+/−^ (gray) and WT (black) relay cells. Detected sEPSCs are indicated by the raster plots above each trace (see also Figure S2). (*b*) Mean sEPSC from the same cells shown in *a*. All sEPSCs detected during 1 min of recording (VGluT1^+/−^*n* = 892; WT *n* = 438) were averaged after aligning by the rising phase. Shaded areas indicate standard deviation. (*c*) Histograms show the mean sEPSC frequency (left) and amplitude (right) for VGluT1^+/−^ (*n* = 8; light) and WT (*n* = 10; dark). Error bars are SEM. Dots show the distribution of values from individual cells. Significant comparisons between WT and VGluT1^+/−^ are indicated: ^*^, *P* < 0.05. (*d*) Example traces from a WT relay cell during the five-pulse stimulation protocol used for variance–mean analysis. Darker trace is the mean of 20–25 repeats. Lighter traces are samples (*n* = 10) of individual repeats. Stimulus artifacts have been cropped for illustrative purposes. (*e*) Mean amplitudes and variance for each stimulus in the train were calculated for each cell and plotted as shown. Single examples of WT (black squares; same cell as in (*d*) and VGluT1^+/−^ (gray dots) cells are shown. These data were fitted with parabolic functions (black and gray, respectively), from which we calculated initial P_R_ and *Q*. (*f*) Summary of the P_R_ and *Q* values determined from the variance–mean analysis of five VGluT1^+/−^ and five WT relay cells (plotted as in *c*). (*g*) Example traces showing the effect of Sr^2+^. Recordings from a WT relay cell following the fifth pulse of a 20-Hz stimulus train in normal (black) and Sr^2+^ (gray) external solutions. The peak of the normal response was clipped for illustrative purposes. During Sr^2+^, multiple aEPSCs (indicated by raster plot above) are visible during the decay phase of the larger synchronous EPSC. Asterix indicates a pair of aEPSCs that were highly overlapping but with sufficient separation to measure both amplitudes. Inset: expanded plots of the six identified aEPSCs after baseline correction (thin lines) and their mean (thick line); dotted line indicates region excluded from average as it was the secondary aEPSC of the pair (^*^). (*h*) Frequency histograms comparing aEPSC amplitudes in WT (left) and VGluT1^+/−^ (right). Each data set was well fit with a gamma probability density function (darker curved lines; WT: *χ*^2^ = 0.0029; VGluT1^+/−^: *χ*^2^ = 0.0062). (*i*) Histogram of the mean aEPSC amplitude for VGluT1^+/−^ (*n* = 6) and WT (*n* = 5) relay cells (plotted as in *c*).

We performed three experiments to determine if P_R_ and/or *Q* might be altered in VGluT1^+/−^ mice. First, we examined spontaneous activity in relay cells from WT and VGluT1^+/−^ mice. For each cell, 1 min of spontaneous activity was analyzed to determine the mean frequency and amplitude of spontaneous(s)-EPSCs (Fig. 7*a*). sEPSCs do not provide direct measures of P_R_ or *Q*; however, changes in these values will be reflected by changes in sEPSC frequency or amplitude, respectively. The shape of the mean sEPSC did not differ between genotypes (Fig. 7*b*). Interestingly, we found a significant genotype difference in sEPSC frequency (*P* = 0.031), while sEPSC amplitude remained unchanged (−9.1 ± 0.42 pA VGluT1^+/−^; −8.9 ± 0.61 pA WT; *P* = 0.81; Fig. 7*c*). sEPSCs occurred at 1.5 times the frequency in VGluT1^+/−^ (12.4 ± 1.34 Hz, *n* = 8) relay cells compared to WT (8.3 ± 1.12 Hz, *n* = 10), suggesting higher P_R_ values in VGluT1^+/−^ mice. This finding is complicated by the fact that sEPSCs result from a mixture of retinal and CT input; however, because the reduced VGluT1 expression should not directly affect VGluT2-expressing retinal synapses, the most likely explanation is that P_R_ is increased at CT synapses. Nevertheless, since we were unable to distinguish retinal-sEPSCs from CT-sEPSCs (Supplemental Fig. S2), the possibility remains that the above result is confounded by changes in retinal-sEPSC frequency or amplitude. To address this issue, we examined P_R_ and *Q* of CT-evoked EPSCs using variance–mean analysis (modified from Saviane and Silver 2006).

To measure variance at different release probabilities, we stimulated CT input with 20-Hz trains of five pulses each, repeated at 30-s intervals (Fig. 7*d*). This protocol produces EPSCs ranging from low P_R_ (EPSC_1_) to high P_R_ (EPSC_5_), similar to the method used by Saviane and Silver (Saviane and Silver 2006), without requiring multiple changes in external calcium concentration. Offline analysis of access and series resistance (calculated from a 5-mV step in holding voltage preceding each train) was used to identify 20–25 consecutive trains with stable values. For each of these trains, the amplitude of each EPSC was individually measured. Mean amplitudes and variance for each stimulus in the five-pulse train were then calculated for each cell and plotted as variance versus mean (Fig. 7*e*). These data were fitted with a parabolic function (Saviane and Silver 2006), from which we calculated P_R_ and *Q* (Fig. 7*f*). Our variance–mean analysis showed that mean *Q* for both VGluT1^+/−^ (*n* = 5) and WT (*n* = 5) relay cells was approximately −5 pA, similar to the smallest sEPSCs detected above and consistent with published values for *Q* at rat CT synapses (Granseth and Lindström 2003). There was no significant difference in *Q* between the genotypes (*P* = 0.85). In addition, this analysis revealed that CT P_R_ in VGluT1^+/−^ was significantly higher (ca. 1.9 times) than in WT (0.098 ± 0.017 and 0.051 ± 0.008, respectively; *P* = 0.038). This value is consistent with the magnitude of the increase in sEPSC frequency we observed.

Finally, to provide a more direct measure of *Q*, we measured the amplitude of asynchronous EPSCs (aEPSCs) during Sr^2+^-mediated persistent release. It has been shown at different types of synapses that replacing extracellular Ca^2+^ with equimolar Sr^2+^ markedly reduces the peak amplitude of evoked EPSCs (due to decreased synchronous vesicle release), while asynchronous vesicle release produces numerous aEPSCs. Since aEPSCs are typically the result of single vesicle release events (Bekkers and Clements 1999; Xu-Friedman and Regehr 2000), their amplitude provides a direct estimate of *Q*. Stimulating CT synapses with single or paired pulses did not produce significant numbers of aEPSCs, so we used 20-Hz trains of five pulses each and measured aEPSCs occurring during or up to 100 ms after the train. Because the aEPSCs produced by this protocol were frequently superimposed in time and occurred during the exponential decay of the initial synchronous EPSC (Fig. 7*g* for sample), aEPSCs were manually identified (based upon exhibiting a clear, rapid rising phase followed by a slower decay) and measured. Careful attention was used to exclude stimulus artifacts, the synchronous EPSCs following each pulse, and other EPSCs that were clearly multivesicular (larger than −20 pA or had multiple rising phases).

We found that Sr^2+^ reduced the amplitude of the synchronous EPSC by 82.7 ± 6.6% in WT and 81.6 ± 17.6% in VGluT1^+/−^ mice (interaction *P* = 0.890, Sr^2+^*P* < 0.0001, genotype *P* = 0.890; two-way RM ANOVA). This effect was partially reversible (% block: 19.4 ± 59.5% in WT and 58.8 ± 18.3% in VGluT1^+/−^). We then analyzed aEPSC amplitudes from five WT and six VGluT1^+/−^ relay cells. Pooled aEPSC amplitude histograms were satisfactorily described by gamma probability density functions with similar shape (*k*) and scale (*θ*) parameters for both genotypes (WT: *k* = 13.5 ± 1.13, *θ* = 0.65 ± 0.06; VGluT1^+/−^: *k* = 12.8 ± 1.56, *θ* = 0.65 ± 0.08; Fig. 7*h*). The distribution of aEPSC amplitudes was not significantly different between genotypes (*P* = 0.997, Kolmogorov–Smirnov test). The mean aEPSC amplitude for VGluT1^+/−^ recordings (−9.0 ± 0.65 pA) was not significantly different from WT (−9.0 ± 0.38 pA; *P* = 0.993; Fig. 7*i*), supporting our previous findings that *Q* is not different between genotypes.

In summary, analysis of spontaneous activity (which could be contaminated by unidentified effects on sEPSCs from retinal synapses), variance–mean analysis of CT-evoked EPSCs (which could be confounded by model-dependent errors), and Sr^2+^-mediated aEPSC amplitude (which cannot assess P_R_ but provides a direct measurement of *Q*) are very different techniques for examining P_R_ and *Q* that do not share the same shortcomings. The variance–mean analysis and sEPSC measurements both agree that P_R_ is almost doubled in VGluT1^+/−^ mice, lending validity to this finding. Furthermore, all three metrics agree that *Q* remains unchanged in VGluT1^+/−^ mice, making this a reliable finding.

## Discussion

We found that VGluT1^+/−^ mice have behavioral impairments in sustained visual-spatial attention and response inhibition. Consistent with this attentional deficit, we found a significant reduction in VGluT1 protein expression at CT synapses in the dLGN. Furthermore, we observed significant impairment of the short-term facilitation typical of CT synaptic connections. One mechanism that likely contributes to the observed decrease in facilitation is an increase in P_R_ in the absence of a measurable change in *Q*. These electrophysiological findings suggest that the dynamic range over which CT synapses can modulate dLGN relay cell excitability is reduced in VGluT1^+/−^ mice. Previous studies have suggested that this dynamic range is critical for the variable regulation of sensory throughput in the thalamus (Ahlsén et al. 1985; Crandall et al. 2015).

### CT Synapses Use VGluT1

Our immunohistochemical examination of dLGN sections confirmed previous findings (Fujiyama et al. 2003; Land et al. 2004; Yoshida et al. 2009) that the patterns of VGluT1 and VGluT2 staining were consistent with CT and OT terminals, respectively, and this was not altered in VGluT1^+/−^ mice. Furthermore, we were able to demonstrate (using the tdTomato-Ntsr1Cre mouse) that VGluT1, but not VGluT2, staining colocalized with tdTomato-labeled CT terminals. Finally, using immuno-EM, we found that VGluT1^+/−^ mice had a 30% reduction in the level of VGluT1 staining within CT profiles.

### Reduction in VGluT1 at CT Terminals does not Change Basal *Q*

A previous study showed that reduced VGluT1 copy number decreases the rate at which vesicles fill with glutamate (Daniels et al. 2006). One possible outcome of this finding would be the release of partially filled vesicles, which could be detected as a reduction in sEPSC amplitude or smaller *Q* values. However, we did not observe reduced sEPSC/aEPSC amplitudes or a reduction in *Q* (variance–mean analysis). This difference between prediction and result could be attributed to three mechanisms. First, *Q* is the amplitude of the postsynaptic response to a single vesicle, not the quantity of glutamate in a single vesicle (quantal content). Thus, homeostatic mechanisms in the postsynaptic membrane (that is, number or sensitivity of the glutamate receptors) might mask changes in the quantal content of synaptic vesicles from VGluT1^+/−^ relative to WT mice. Second, research using cultured hippocampal neurons has demonstrated that partially filled glutamatergic vesicles are more difficult to release (Herman et al. 2014). Thus, spontaneous or evoked EPSCs are unlikely to result from partially filled vesicles, unless the pool of “full” vesicles is insufficient. Third, the techniques we used to calculate *Q* were conducted under conditions where vesicles were not depleted faster than they could be refilled. In summary, vesicle-refilling rate is predicted to have little influence on sEPSC amplitude or *Q* values during low-frequency stimulation, when the pool of “full” vesicles is sufficient to allow recycled vesicles the additional time needed to refill. In order to observe whether VGluT1 expression levels had an impact on vesicle-refilling rates, we instead examined prolonged, high-frequency stimulation.

### A Reduced Neurotransmitter-Refilling Speed Becomes Apparent During Intense Activity

During train stimulation, we observed a significantly decreased latency to depression and stronger levels of depression in VGluT1^+/−^ mice, relative to WT. These differences cannot be explained by changes in vesicle numbers, as EM experiments showed that synapse size and vesicle density were normal in CT terminals of VGluT1^+/−^ mice. Instead, these differences likely arise from the observed decrease in VGluT1 expression. With fewer transporters per vesicle, the vesicle-refilling rate should be reduced.

Once stimulation frequency exceeds the refilling rate, partially filled vesicles will be brought to the active zone. These partially filled vesicles might then be prevented from fusing until “full” (as per [Herman et al. 2014]) or be released with a lower glutamate concentration. Both situations would lead to smaller EPSCs being recorded in the postsynaptic relay cell, as we observed during the later stages of train stimulation (particularly at 20 Hz). Unfortunately, using slice preparations, we cannot distinguish which mechanism leads to this earlier depression. Techniques that allow simultaneous recording of EPSCs (in relay cells) and vesicle fusion (in CT terminals) would be needed to resolve this difference.

### Reduction in VGluT1 at CT Terminals Increases Initial P_R_

**Figure 8:**
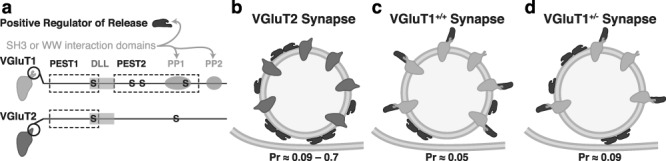
VGluT1 braking hypothesis. (*a*) Comparison of the C-terminal tails of VGluT1 and 2. The C terminal is cytosolic and contains multiple interaction domains: PEST (proline, glutamic acid, serine and threonine residue rich), DLL (dileucine-like internalization motif), and S (serine phosphorylation site). VGluT1, in particular, has two PP (polyproline) regions that contain multiple binding sites for SH3 (*Src* homology 3) and WW-domain-containing proteins. Binding of PP regions on VGluT1 to an unidentified positive regulator of release (PRR), which contains SH3 or WW domains, could underlie differences in P_R_ associated with the two VGluT isoforms, as illustrated in *b* and *c*. P_R_ values for VGluT2 taken from hippocampal neurons misexpressing VGluT2 (low) and retinothalamic synapses (high) (Weston et al. 2011; Budisantoso et al. 2012). VGluT1 P_R_ values from Fig. 7f. PRR that is bound by VGluT1 is not able to positively regulate release of synaptic vesicles, thus P_R_ is reduced (*c*). In this sense, VGluT1 is acting like a brake on the normal release machinery. In VGluT1^+/−^ mice (*d*), the number of VGluT1 proteins per vesicle is reduced, effectively increasing the amount of unbound PRR and allowing higher P_R_.

VGluT1^+/−^ mice displayed a significant increase in P_R_ that could not be attributed to changes in active zone size or number of vesicles. Instead, our data suggest that VGluT1 has an inherent effect on how efficiently vesicles can be released. VGluT1 has been shown to interact with a number of presynaptic proteins via two C-terminal polyproline domains (PP1 and PP2; Fig. 8*a*) that are not present in VGluT2 (Santos et al. 2014), including an interaction with endophilin that was found to reduce P_R_ (Weston et al. 2011). In addition to endophilin’s previously identified roles in endocytosis (Milosevic et al. 2011), endophilin A1 was recently found to enhance the efficiency of exocytosis. This exocytotic function of endophilin was inhibited by VGluT1 binding (Weston et al. 2011). In other words, VGluT1 could reduce P_R_ (relative to VGluT2) by buffering endophilin activity. While we have not directly examined the presence of endophilin at CT synapses, the Allen Brain Transcriptome Database shows high levels of SH3GL2 (the gene encoding endophilin A1) expression in layer 6 CT neurons (Tasic et al. 2016).

Given these previous findings and our results, we propose that at CT synapses, VGluT1 normally inhibits a presynaptic protein that is a positive regulator of release (PRR; Fig. 8*b*,*c*). In this sense, VGluT1 normally acts as a brake to slow vesicle release, producing the extremely low initial P_R_ that is characteristic of this synapse. The 30% reduction in VGluT1 levels in VGluT1^+/−^ mice would, therefore, provide less braking activity (Fig. 8*d*) resulting in the observed increase in initial P_R_ (Fig. 7*f*).

This braking model also suggests one molecular mechanism that may contribute to facilitation at CT synapses. Activity-dependent reduction in the binding affinity between VGluT1 and the PRR (maybe via phosphorylation of the VGluT1 C terminal (Santos et al. 2014) would “disengage the brake” by increasing the unbound-PRR concentration and thus the P_R_. An upper limit for how much this mechanism can enhance P_R_ is set by saturation of the unbound-PRR effect. Thus, differences in the initial amount of VGluT1 braking can alter the observed level of facilitation. Since there is less brake to disengage in VGluT1^+/−^ mice, this would explain the observed reduction in the slow phase of facilitation (under conditions when vesicle-refilling speed is not rate limiting; that is, paired-pulse data and initial portions of train stimuli). This hypothesis requires additional study to identify possible PRRs that interact with VGluT1 and then determine which interactions can be influenced by activity-dependent mechanisms.

Taken together, the results above suggest that reduced VGluT1 expression impairs short-term facilitation at CT synapses via two primary mechanisms: (1) reduction of a braking mechanism that typically keeps initial P_R_ low and contributes to facilitation and (2) reduced vesicle refilling rates that produce earlier depression during prolonged activation. The combined hit of increased initial P_R_ and decreased facilitation means that VGluT1^+/−^ mice have a significantly reduced dynamic range for CT gain regulation of dLGN relay cells.

### The Reduction in Short-Term Facilitation at CT Terminals Impairs Visual-Spatial Attention

A recent study found that facilitation at CT to relay cell synapses is an important component of dynamic gain regulation in the thalamus, determining the level of sensory throughput and synchronization with cortical gamma oscillations (Crandall et al. 2015). Thus, the severe reduction in the dynamic range for CT facilitation that we observed in VGluT1^+/−^ mice would be expected to impair modulation of sensory throughput and synchronization with cortical oscillations. Regulation of gain in the thalamus and/or synchronization with cortical oscillations have been proposed as mechanisms by which the cortex provides attentional regulation of sensory information passing through the thalamus (Briggs and Usrey 2008; McAlonan et al. 2008; Saalmann and Kastner 2009). Our finding that mice with reduced VGluT1 expression had impairments in sustained visual-spatial attention would support this hypothesis. The next step to confirm this relationship would be to examine sensory throughput and synchronization in the thalamus of VGluT1^+/−^ mice.

The mice used in this study have global VGluT1 hemizygosity. Thus, other VGluT1-expressing neurons could contribute to the observed deficit in visual-spatial attention. In line with this, we observed impairment in response inhibition, which suggests that additional neural networks (likely involving prefrontal cortex) are disrupted in the VGluT1^+/−^ mice. Since the disrupted networks must involve VGluT1-expressing pathways, it is tempting to speculate that a similar reduction in short-term facilitation at other modulatory synapses (like those between prefrontal cortex and mediodorsal thalamus [Rovó et al. 2012; Acsády 2017; Bolkan et al. 2017; Schmitt et al. 2017] or nucleus accumbens [Fujiyama et al. 2004; Granseth et al. 2015]) also contributes to this deficit. However, further experiments would be necessary to verify this hypothesis.

## Supplementary Material

Suppl_Fig1_bhz204

Suppl_Fig2_bhz204
